# Peaked T - Waves and Sinus Arrhythmia Before Prolonged Sinus Pauses and Atrioventricular Block in Guillain-Barre Syndrome

**Published:** 2007-10-22

**Authors:** Adriano Tonelli, Atul Khasnis, George S Abela

**Affiliations:** 1 Internal Medicine Resident, Department of Internal Medicine, Michigan State University, East Lansing, Michigan; 2 Chief of the Cardiology Division, Department of Internal Medicine, Michigan State University, East Lansing, Michigan

**Keywords:** Guillain-Barre Syndrome, Autonomic dysfunction, Arrhythmia, Heart block

An 18-year-old woman presented with a rapidly progressive ascending flaccid paralysis and numbness requiring mechanical ventilation after an upper respiratory infection. Guillain-Barre Syndrome was diagnosed based on albumino-cytologic dissociation in the cerebrospinal fluid (elevated proteins with normal white blood cell count) and a demyelinating sensory-motor polyneuropathy pattern on the electromyogram. She was initially treated with intravenous immunoglobulin, but later required plasmapheresis for non-resolution of symptoms.

Her initial electrocardiogram was normal (Figure 1). Her hospital course was complicated by the development of sinus tachycardia with wide fluctuations in the heart rate (Figure 2). Starting on the 12th day, she had repeated episodes of severe bradycardia (sinus pauses and paroxysmal atrioventricular block) with prolonged episodes of asystole up to 5.7 seconds in duration (Figure 3) and episodes of non-sustained ventricular tachycardia (Figure 4). These arrhythmias occurred spontaneously, without relation to vagal maneuvers such as endotracheal suctioning. Serum electrolytes, including potassium, magnesium and calcium were within normal limits. Immediately before the development of the bradyarrhythmias, we saw new peaked T waves (Figure 2), shortening of the corrected QT interval (Figure 2), variation of the amplitude and possibly axis of the P waves (Figure 3), associated with marked sinus arrhythmia and heart rate variability (Figure 5). A temporary pacemaker was initially placed, but she later required implantation of a permanent pacemaker for continued severe bradyarrhythmia for three days. The pacemaker was programmed as VVI mode at 40 beats per minute. After the sixth week, the variability of the heart rate markedly decreased, most likely expressing resolution of the autonomic imbalance.

Serious cardiac rhythm disturbances appear frequently in severely ill patients with Guillain-Barre syndrome. These events have been attributed to autonomic dysfunction, which is common in patients with Guillain-Barre syndrome and manifests as inappropriate sinus tachycardia, reduced R-R interval variation, postural hypotension, excessive sweating and reactive hypertension [1,2]. Paroxysms of parasympathetic activity with wide fluctuations of heart rate and blood pressure have been noted to precede deaths in fatal cases [2,3]. Periods of bradycardia and asystole have been observed during tracheal suctioning [1,4,5]. Severe arrhythmias are seen more commonly in patients with severe disease requiring mechanical ventilation [1,5]. Continuous electrocardiographic monitoring during the acute phase of the disease is important for early identification of patients who will develop severe arrhythmias [1,3,5]. The management of severe bradyarrhythmias in patients with Guillain-Barre syndrome is based on the insertion of a temporary pacemaker as these events are usually transient. Few cases require the implantation of a permanent pacemaker [4].

Our patient had inappropriate sinus tachycardia, marked sinus arrhythmia and interestingly pronounced peaked T waves, corrected QT interval and variation in the morphology of the P waves immediately preceding the development of sinus pauses and paroxysmal atrioventricular block, demonstrating the entire spectrum of autonomic dysfunction. Changes in the T and P wave morphology and QT intervals that fail to prolong in the presence of bradycardia (also referred as paradoxical failure of the QT prolongation) have been reported in association with variation in the autonomic tone during tilt table testing [6-8], however, to the best of our knowledge, they have not been described in patients with Guillain-Barre syndrome. The electrocardiographic findings seen in our patient may be explained by a state of increased sympathetic activation with accentuated antagonism of sympathetic activity by a sudden vagal discharge with subsequent marked parasympathetic manifestations [8. A relatively less antagonism of the sympathetic activity at the level of the ventricles, due to less vagal innervation, may explain why the QT does prolong in the presence of bradycardia [8].

The development of the aforementioned electrocardiographic variations may identify patients with GBS and autonomic lability who may require the implantation of a temporary pacemaker.

## Figures and Tables

**Figure 1:**
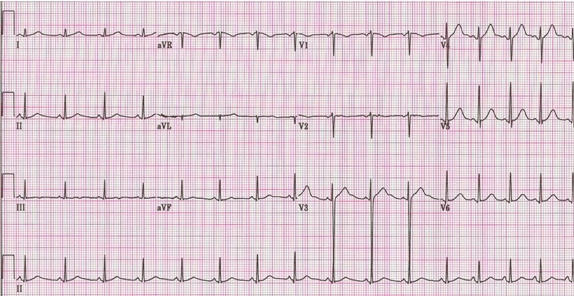
Baseline electrocardiogram.  The QT interval and the corrected QT interval are 360 ms and 450 ms respectively.

**Figure 2:**
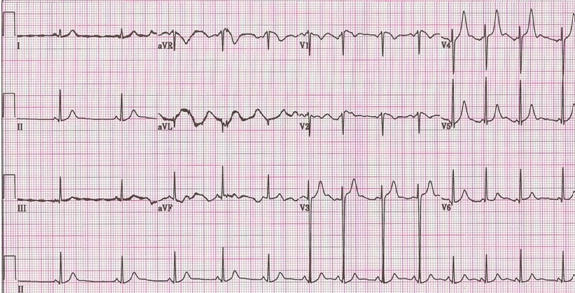
Electrocardiogram just before the occurrence of severe bradyarrhythmias showing peaked T waves on the precordial leads. Leads aVR , aVL and aVF have artifact. The QT interval and the corrected QT interval are 320 ms and 405 ms respectively.

**Figure 3:**
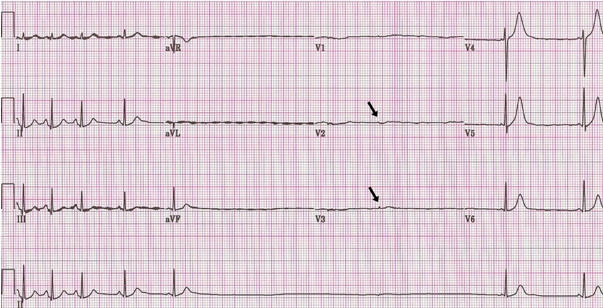
Prolonged asystole (5.4 sec) revealing a blocked P wave (arrows) and peaked T waves. Note the different P wave morphology before and immediately after the asystole.

**Figure 4:**
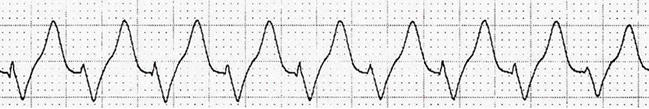
Non-sustained ventricular tachycardia recorded in a telemetry strip. (150 beats per minute).

**Figure 5:**
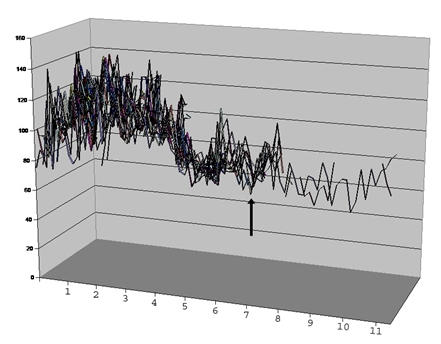
Heart rate chart, showing tachycardia with significant heart rate variability in the first 2 weeks after the admission, period when the patient developed severe bradyarrhythmias. Tick marks in the y-axis represent the heart rate in beats per minute. Tick marks in the x-axis represent weeks. The pacemaker was programmed at 40 beats per minute and does not explain the decrease in heart rate variability after the sixth week of hospitalization (arrow), most likely explained by a normalization of the autonomic balance.
